# Substrate-Dependent Inhibition of the Human Organic Cation Transporter OCT2: A Comparison of Metformin with Experimental Substrates

**DOI:** 10.1371/journal.pone.0136451

**Published:** 2015-09-01

**Authors:** Kristina Hacker, Renke Maas, Johannes Kornhuber, Martin F. Fromm, Oliver Zolk

**Affiliations:** 1 Institute of Experimental and Clinical Pharmacology and Toxicology, Friedrich-Alexander-Universität Erlangen-Nürnberg, Erlangen, Germany; 2 Department of Psychiatry and Psychotherapy, Friedrich-Alexander-Universität Erlangen-Nürnberg, Erlangen, Germany; 3 Institute of Pharmacology of Natural Products & Clinical Pharmacology, Ulm University, Ulm, Germany; Hungarian Academy of Sciences, HUNGARY

## Abstract

The importance of the organic cation transporter OCT2 in the renal excretion of cationic drugs raises the possibility of drug-drug interactions (DDIs) in which an inhibitor (perpetrator) drug decreases OCT2-dependent renal clearance of a victim (substrate) drug. In fact, there are clinically significant interactions for drugs that are known substrates of OCT2 such as metformin. To identify drugs as inhibitors for OCT2, individual drugs or entire drug libraries have been investigated *in vitro* by using experimental probe substrates such as 1-methyl-4-phenylpyridinium (MPP^+^) or 4–4-dimethylaminostyryl-N-methylpyridinium (ASP^+^). It has been questioned whether the inhibition data obtained with an experimental probe substrate such as MPP^+^ or ASP^+^ might be used to predict the inhibition against other, clinical relevant substrates such as metformin. Here we compared the OCT2 inhibition profile data for the substrates metformin, MPP^+^ and ASP^+^. We used human embryonic kidney (HEK 293) cells stably overexpressing human OCT2 as the test system to screen 125 frequently prescribed drugs as inhibitors of OCT2-mediated metformin and MPP^+^ uptake. Data on inhibition of OCT2-mediated ASP^+^ uptake were obtained from previous literature. A moderate correlation between the inhibition of OCT2-mediated MPP^+^, ASP^+^, and metformin uptake was observed (pairwise *r*_s_ between 0.27 and 0.48, all *P* < 0.05). Of note, the correlation in the inhibition profile between structurally similar substrates such as MPP^+^ and ASP^+^ (Tanimoto similarity *T* = 0.28) was even lower (*r*_s_ = 0.27) than the correlation between structurally distinct substrates, such as ASP^+^ and metformin (*T* = 0.01; *r*_s_ = 0.48) or MPP^+^ and metformin (*T* = 0.01; *r*_*s*_ = 0.40). We identified selective as well as universal OCT2 inhibitors, which inhibited transport by more than 50% of one substrate only or of all substrates, respectively. Our data suggest that the predictive value for drug-drug interactions using experimental substrates rather than the specific victim drug is limited.

## Introduction

The kidneys play an important role in the elimination of drugs. In a recent analysis of clinical elimination data for 391 drugs, Varma *et al*. showed that especially hydrophilic ionized compounds show net renal secretion [1]. About 40% of all prescribed drugs and even two-thirds of all psychotropic drugs are cationic at physiological pH [1, 2]. The basic cellular model of the renal tubular secretion of these organic cations includes the sequential activity of a basolateral “entry step” from the blood into the renal proximal tubule cells, which involves an electrogenic organic cation transporter, and an apical “exit step” from the cells to the tubular filtrate, which is mediated by electroneutral organic cation—proton exchangers, such as multidrug and toxin extrusion transporters (MATEs). In humans, the basolateral step in this process is dominated by the activity of the multispecific organic cation transporter OCT2 (*SLC22A2*) [3, 4].

The important role of the organic cation transporter OCT2 in the renal excretion of cationic drugs raises the possibility of drug-drug interactions (DDIs) in which an inhibitor (perpetrator) drug decreases OCT2-dependent renal clearance of a victim (substrate) drug. In fact, there are several clinical examples of drug-drug interactions mediated by inhibition of cation transporters. It is well known that the concomitant use of the potent OCT / MATE inhibitor cimetidine reduces the renal clearance of drugs which are organic cations, such as procainamide, ranitidine, triamterene, metformin, and flecainide by competing for active tubular secretion in the proximal tubule of the kidney [5]. In addition, the inhibition of renal OCT2-mediated drug transport by cimetidine or verapamil can decrease drug accumulation within the kidney, thereby reducing the nephrotoxicity associated with the use of the cationic anticancer drug cisplatin [6]. These observations suggest that OCT2-mediated drug-drug interactions are clinically relevant.

Hundreds of xenobiotics and drugs that potentially inhibit OCT2 were tested in the past [7, 8]. These *in vitro* screens have led to the identification of several potent OCT2 inhibitors although the identification among all approved and marketed drugs is still incomplete. Most of the *in vitro* screens were performed with non-drug/experimental probe substrates such as 1-methyl-4-phenylpyridinium (MPP^+^) or 4–4-dimethylaminostyryl-N-methylpyridinium (ASP^+^), because the compounds were recommended for *in vitro* tests by the U S Food and Drug Administration (MPP^+^), or the compounds exhibit native fluorescence (ASP^+^) or are radiolabelled and therefore can easily be used in high throughput assays.

It has been proposed that OCT2, like many other polyspecific drug transporters, has multiple binding sites and that substrates and inhibitors may interact with one or more of these sites, perhaps simultaneously [9, 10]. Whether a compound is an OCT2 inhibitor or not may, therefore, depend on the respective substrate. In fact, recent studies have noted an influence of a substrate on the inhibition profile of perpetrator drugs. Belzer *et al*. for example compared IC_50_ values obtained for a set of structurally distinct inhibitors against OCT2-mediated transport of structurally distinct substrates and concluded that cationic drugs were generally approximately 10 times more effective inhibitors of OCT2-mediated metformin transport than of MPP^+^ transport. The findings suggest that there is a correlation between the inhibition profiles obtained with different probe substrates. The inhibition profile obtained with an experimental probe substrate such as MPP^+^ might thus be used to predict the inhibition profile against another substrate such as metformin, provided a proportionality factor is considered.

The primary aim of this study was to test whether the inhibitory effects of drugs on OCT2-mediated transport strongly correlate between structurally distinct substrates, or whether some drugs potently inhibit the OCT2-dependent transport of one substrate whereas the transport of another substrate is not affected (selective inhibition of OCT2-mediated transport of specific substrates). Therefore, we screened a library of 125 frequently prescribed, structurally diverse drugs for their ability to inhibit OCT2. We used metformin and MPP^+^ as chemically different probe substrates in our *in vitro* screening approach. Additionally, we compared our data with an external data set for the inhibition of OCT2-mediated ASP^+^ uptake [7].

Secondary aims were to identify novel potent inhibitors of OCT2-mediated transport and to estimate whether these drugs might be clinically relevant perpetrator drugs interacting with victim/substrate drugs such as metformin. To reach the latter goal, we determined IC_50_ values of drugs identified as potent inhibitors and calculated the ratio of the unbound peak plasma concentration (C_max,u_) and the half-maximal inhibitory concentration (IC_50_ value) of the respective drugs. C_max,u_ / IC_50_ > 0.1, which is used by the FDA as a criterion for the need for further *in vivo* drug interaction studies, was used as a threshold to define drug-drug interactions possibly relevant in clinical practise.

## Materials and Methods

### Drugs

Benperidol, isosorbide dinitrate, perazine, rivastigmine and xipamide were ordered from Chemos (Regenstauf, Germany). Aripiprazole, alendronate, candesartan, duloxetine, felodipine, hydrochlorothiazide, irbesartan, losartan, olmesartan and trospium chloride were purchased from Molekula (Nienburg, Germany). Melperone was from Tocris Bioscience (Bristol, UK), doxycycline was from Pfizer (Berlin, Germany), bisoprolol, ezetimibe and pravastatin were from BioTrend (Cologne, Germany). All other drugs were ordered from Sigma Aldrich (Taufkirchen, Germany). All compounds were of analytical grade and of at least 95% purity. Most stock solutions were prepared with water or DMSO as solvents. Drugs insoluble in DMSO or water were dissolved in ethanol, methanol or 0.1 N HCl. Immediately before the experiments appropriate amounts of the stock solution were diluted with uptake buffer (pH 7.3, for composition see below) so that the concentration of the solvent generally did not exceed 1‰ in the final working solution. In rare cases the poor solubility of the respective compound required final solvent concentrations higher than 1‰ but less than 5‰. To exclude effects of the solvent, solvent control experiments were performed in parallel.

### Transport Assays in HEK-OCT2 Cells

**Fig 1:**
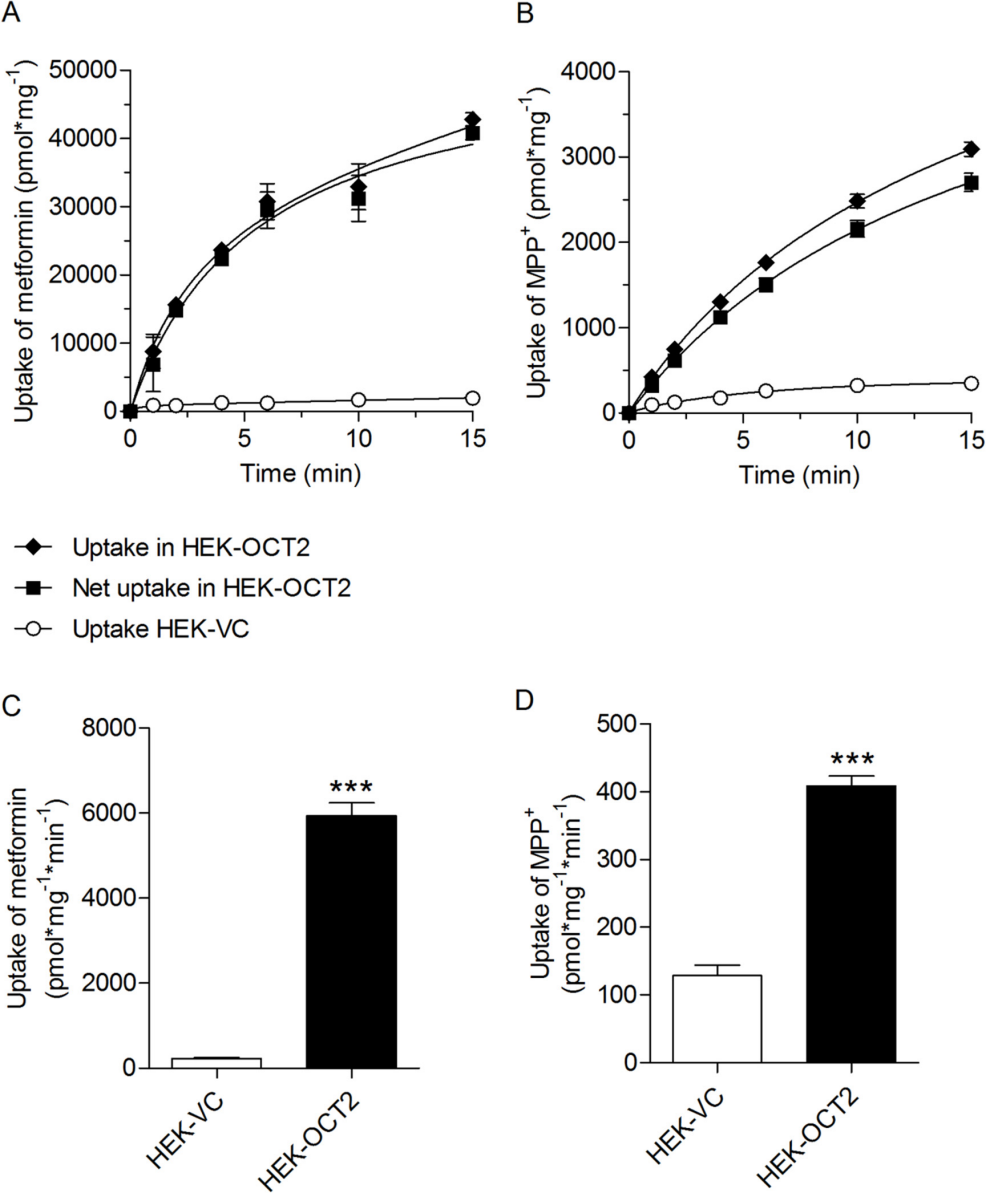
OCT2-mediated metformin and MPP^+^ uptake. Time-dependent uptake of metformin (A, 1000 μM) and MPP^+^ (B, 50 μM) into OCT2-expressing HEK-cells and corresponding vector controls. Uptake of metformin (C) and MPP^+^ (D) in HEK-VC and HEK-OCT2 cells after three minutes. Data are presented as the mean ± standard error (4–8 experiments each on two to four separate days, i.e., *n* = 8–29).

For the transport assays HEK cell lines stably transfected with human OCT2 (HEK-OCT2) or the vector only (HEK-VC) were used. The HEK-OCT2 cell line was established and characterized previously [8, 11]. HEK-OCT2 and HEK-VC cells were seeded in poly-D-lysine (Sigma Aldrich, Taufkirchen, Germany) coated 48-well-plates at a density of 1.2 x 10^5^ cells/well. After incubation at 37°C and 5% CO_2_ for 48 h medium was replaced by pre-warmed (37°C) uptake buffer (142 mM NaCl, 5 mM KCl, 1 mM K_2_HPO_4_, 1.2 mM MgSO_4_, 1.5 mM CaCl_2_, 5 mM glucose and 12.5 mM HEPES, pH 7.3) with or without the test drug. The assay was started by addition of [^14^C]metformin (92.7 mCi / mmol, Moravek Biochemicals, Brea, California) or [^3^H]MPP^+^ (80 Ci / mmol, American Radiolabeled Chemicals, St Louis, MO). In a total incubation volume of 125 μL the final concentrations of the test drugs were 20 or 200 μM. Final substrate concentrations were 1000 μM for [^14^C]metformin and 50 μM for [^3^H]MPP^+^. Cells were incubated at 37°C for 3 min, a time-point well before kinetics of OCT2-mediated uptake of MPP^+^ and metformin reached steady-state (Fig 1). Uptake was stopped by washing the cells three times with ice-cold uptake buffer. Afterwards, cells were lysed with 5 mM Tris buffer (pH 7.3) containing 0.1% Triton X-100. The intracellular accumulation of radioactivity was determined by liquid scintillation counting (PerkinElmer, Rodgau-Jügesheim, Germany) and protein concentration of each lysate was measured with bicinchonic acid assay (BCA Protein Assay Kit, Thermo Fisher Scientific, Waltham, MA, USA). We performed two to eight experiments each on at least two separate days, i.e., *n* = 4–16. The OCT2-mediated net uptake of [^14^C]metformin and [^3^H]MPP^+^ was determined as the difference in substrate uptake between HEK-OCT2 and HEK-VC cells. The percentage of uptake inhibition was calculated as: Inhibition (%) = 100 –(*V* / *V*_0_ * 100), where *V* and *V*_0_ are the net uptake rates with and without test drug.

To investigate whether structurally distinct substrates may display different inhibitory profiles for the same set of test drugs, the inhibition values for one substrate were plotted against the inhibition values for the other substrate. Spearman correlation tests were used to determine the strength of the monotonic relationships *r*_*s*_ between the two variables. Bland-Altman analysis was used to quantify the level of agreement. Each of the 125 drugs was represented on the Bland-Altman graph by assigning the mean of the two measurements (% inhibition of OCT2-mediated uptake of MPP^+^ and metformin) as the abscissa value, and the difference between the two values as the ordinate value.

For a further in-depth investigation of the substrate dependence of OCT2-inhibition, our *in vitro* data were compared with data published by Kido *et al*. [7], who studied the inhibition of OCT2-mediated ASP^+^ transport. Kido *et al* used a similar experimental screening approach, namely a drug library tested at 20 μM in HEK 293 cells stably overexpressing OCT2.

### IC_50_ Determination

Experimental half-maximal inhibitory concentrations (IC_50_ values) were measured as the substrate uptake in the presence of increasing concentrations of test drugs. These experiments were performed as described above (three experiments each on two separate days, i.e., *n* = 6). IC_50_ values were calculated with GraphPad Prism 5.0 (GraphPad Software, San Diego, CA, USA). IC_50_ was estimated by a sigmoidal inhibition model and was fit to the equation *V = V*_0_ */ (*1 *+ (I /* IC_50_ *)* ^*n*^ *)* by nonlinear regression. *V* is the net uptake in the presence of the inhibitor, *V*_0_ is the net uptake in the absence of the inhibitor, *I* is the concentration of the inhibitor and *n* is the slope.

### Tanimoto Similarity

Structural similarity between the OCT2 substrates metformin, MPP^+^ and ASP^+^ was investigated. Therefore, the Tanimoto pairwise similarity coefficient was calculated as an accepted distance metric for topology-based chemical similarity using ChemMine [12]. The pairwise Tanimoto similarity between compounds A and B is described as follows: *T*_A,B_ = c / (a + b − c), where c represents the number of bits set to 1 common to the structural fingerprints of compounds A and B and a and b represent the number of bits set to 1 in fingerprints of A and B, respectively. The Tanimoto coefficient (*T*) has a range from 0 to 1 with higher values indicating greater similarity than lower ones.

### Statistical analysis

Comparison of metformin or MPP^+^ uptake in HEK-VK and HEK-OCT2 cells was performed using the unpaired two-sample t test. Significant inhibition of OCT2 mediated metformin or MPP^+^ uptake was determined by the one-sample t test. Univariate relationships were tested by Spearman’s correlation coefficient. Data were presented as means ± standard error of the mean. A value of *P* < 0.05 was considered statistically significant.

A standard multiple regression was performed to assess the ability of the molecular descriptors topological surface area (TPSA), number of aromatic rings, net charge (at pH 7.4), distribution coefficient (logD at pH 7.4), and molecular weight to predict the percent inhibition of OCT2-mediated MPP^+^ or metformin uptake. We used the inhibition data set generated with 20 μM drug concentrations. The molecular descriptors (independent variables) were calculated with Marvin (Chemaxon, Budapest, Hungary) and ChemMine [12]. They were selected based on previously published studies on the structure-activity relationship [7, 8, 13]. Multiple regression analysis was performed with SPSS Statistics, version 21 (IBM Corporation, Armonk, NY).

## Results

### Inhibition of OCT2-Mediated Metformin and MPP^+^ Uptake

**Fig 2:**
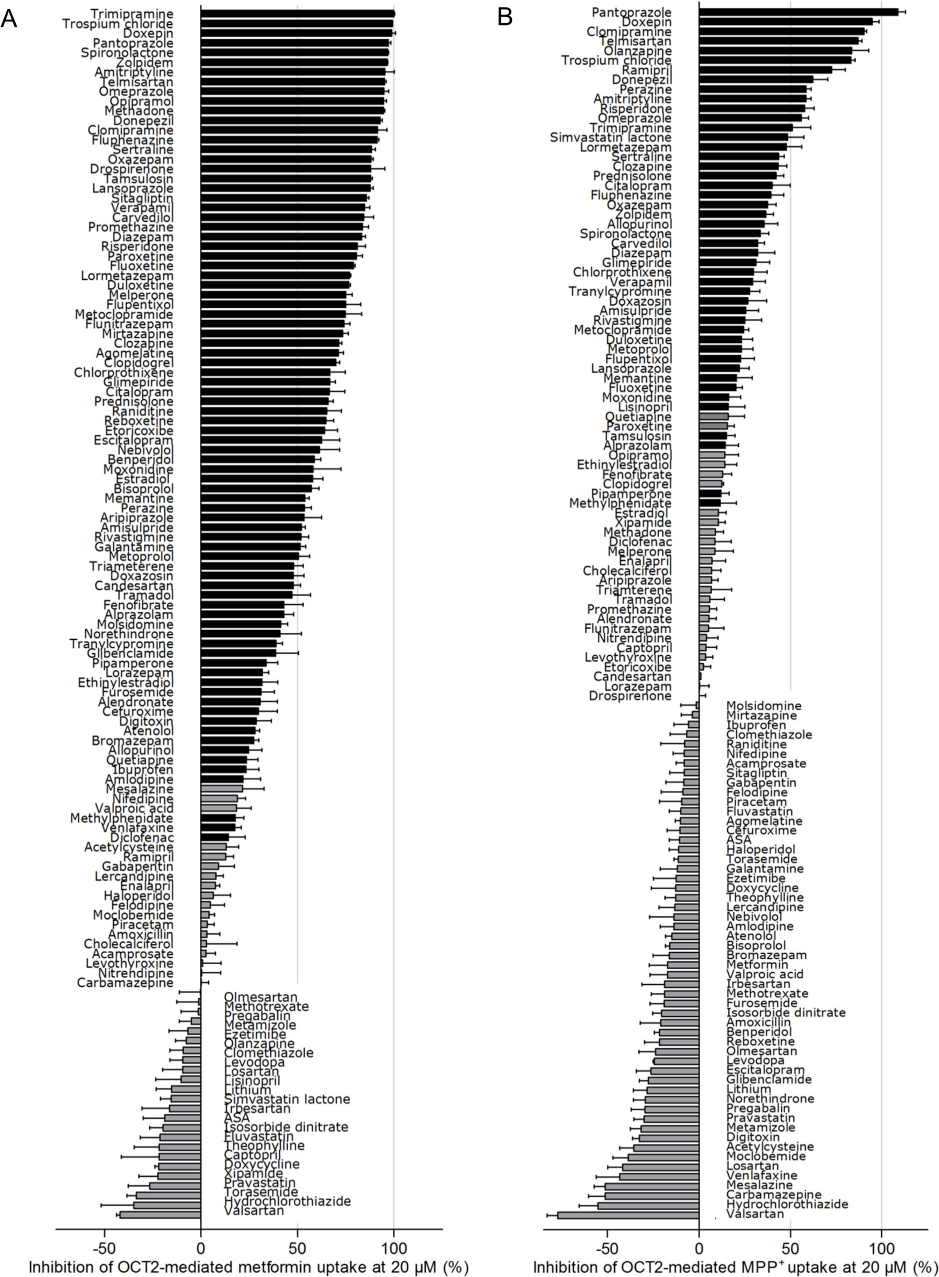
Inhibitors of OCT2-mediated metformin (1000 μM) and MPP^+^ (50 μM) transport identified in a screen of 125 drugs most commonly prescribed in Germany. (A) Inhibition of metformin transport. (B) Inhibition of MPP^+^ transport. Each bar represents one compound tested in HEK-OCT2 cells at a concentration of 20 μM. Bars showing a statistically significant (one-sample t test) inhibition are shaded in black. Data are presented as the mean +/- standard error (at least two experiments each on two or more separate days, i.e., *n* = 4–15). Negative inhibition values indicate enhanced OCT2-dependent substrate uptake in cells incubated with the respective test drug compared with vehicle-treated cells; ASS, acetylsalicylic acid.

OCT2-mediated uptake of metformin (1000 μM) and MPP^+^ (50 μM) in HEK-OCT2 cells was time-dependent and uptake reached steady-state after approximately 15 min (Fig 1A and 1B). For the subsequent transport inhibition assays an incubation time of 3 minutes was chosen, which was within the initial almost linear upstroke of the time uptake curve. In the absence of an inhibitor the uptake ratio was 26, i.e., the uptake and accumulation of metformin in OCT2 cells was 26—times higher compared with vector control cells (5946 ± 294.0 pmol * mg^-1^ * min^-1^ vs. 227 ± 22.1 pmol * mg^-1^ * min^-1^, Fig 1C). The ratio of MPP^+^ uptake in OCT2-HEK and vector control cells was 3.2 (Fig 1D). The drug library was screened at concentrations of 20 μM (Fig 2) and 200 μM (Supplemental Data, S1 and S2 Figs). Data are presented as the percentage inhibition of the OCT2-mediated uptake of metformin or MPP^+^.

At 20 μM, eighty-three of the 125 tested drugs (66%) significantly inhibited OCT2-dependent metformin uptake. Fifty-seven of these drugs inhibited OCT2-mediated metformin transport by more than 50% (Fig 2A). At 200 μM the inhibition profile of the drug library was shifted towards a stronger inhibition of metformin transport, suggesting concentration dependency (S1 Fig). The most potent inhibitors of OCT2-mediated metformin uptake included trimipramine, trospium chloride, doxepin, and pantoprazole.

45 of 125 drugs significantly inhibited MPP^+^ uptake when a drug concentration of 20 μM was used. Thirteen drugs most potently inhibited MPP^+^ transport by more than 50% (Fig 2B), suggesting that their IC_50_ values were less than 20 μM. Pantoprazole, doxepin, clomipramine and olanzapine were the most potent inhibitors of OCT2-mediated MPP^+^ uptake. In general, inhibition of OCT2-mediated MPP^+^ uptake was basically concentration dependent, because the inhibition profile of the drug library was shifted towards stronger inhibition with 200 μM vs. 20 μM concentrations (S2 Fig). Some of the investigated drugs (e.g., hydrochlorothiazide) stimulated OCT2-mediated uptake of metformin and/or MPP^+^ (Fig 2, S1 and S2 Figs). The underlying mechanism of this stimulating effect, which has also been observed by others [7], is currently unknown.

### Substrate-Dependent Differences in the Inhibitory Profiles

Prototypical substrates used to identify inhibitors of OCT2 *in vitro* include the structurally diverse compounds MPP^+^, ASP^+^, and metformin. Tanimoto pairwise similarity scores between metformin and MPP^+^ and between metformin and ASP^+^ were each 0.01, indicating structural dissimilarity. Although MPP^+^ and ASP^+^ were structurally less diverse, the Tanimoto score of *T* = 0.28 did not indicate significant, above-average structural similarity between these two OCT2 substrates.

**Fig 3:**
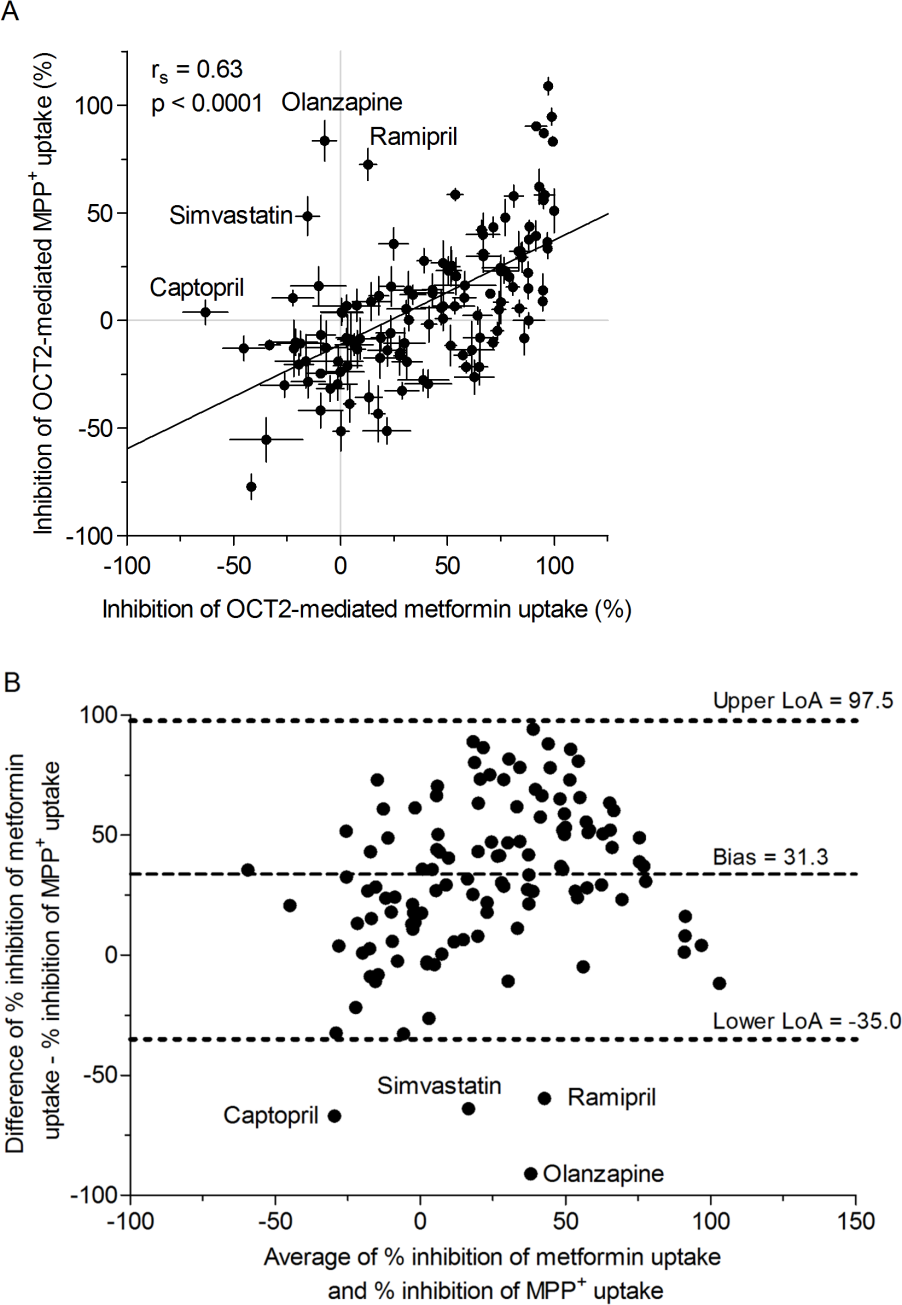
Substrate-dependent differences in the inhibitory profiles and concentration-dependent inhibition of metformin uptake. (A) Correlation analysis between inhibition of OCT2-mediated metformin and inhibition of OCT2-mediated MPP^+^ uptake in HEK-OCT2 cells. Drugs were tested at 20 μM. Substrate concentrations were 1000 μM for metformin and 50 μM for MPP^+^ (data are presented as the mean ± standard error, Spearman's rank-order correlation test). (B) Bland-Altman plot (bias and limits of agreement) for the inhibition of OCT2-mediated metformin and MPP^+^ uptake. Average of % inhibition of metformin uptake and % inhibition of MPP^+^ is plotted against the difference between % inhibition of metformin uptake and % inhibition of metformin uptake, LoA, limits of agreement. Simvastatin, simvastatin lactone

To investigate whether the observed inhibitory potency of a drug differs with the substrate used in the experiments (metformin *vs*. MPP^+^), quantitative measures for the inhibition of metformin and MPP^+^ uptake were correlated for each drug and tested using the Spearman's rank correlation. At an inhibitor concentration of 20 μM a moderate (*r*_*s*_ = 0.63) but significant correlation (*p* < 0.0001) between the inhibition of OCT2-mediated metformin and MPP^+^ uptake was observed (Fig 3A). The Bland-Altman plot (Fig 3B), which shows bias and limits of agreement between the inhibition of OCT2-mediated metformin and MPP^+^ uptake, indicates a systematic difference between both substrates (fixed bias).

**Fig 4:**
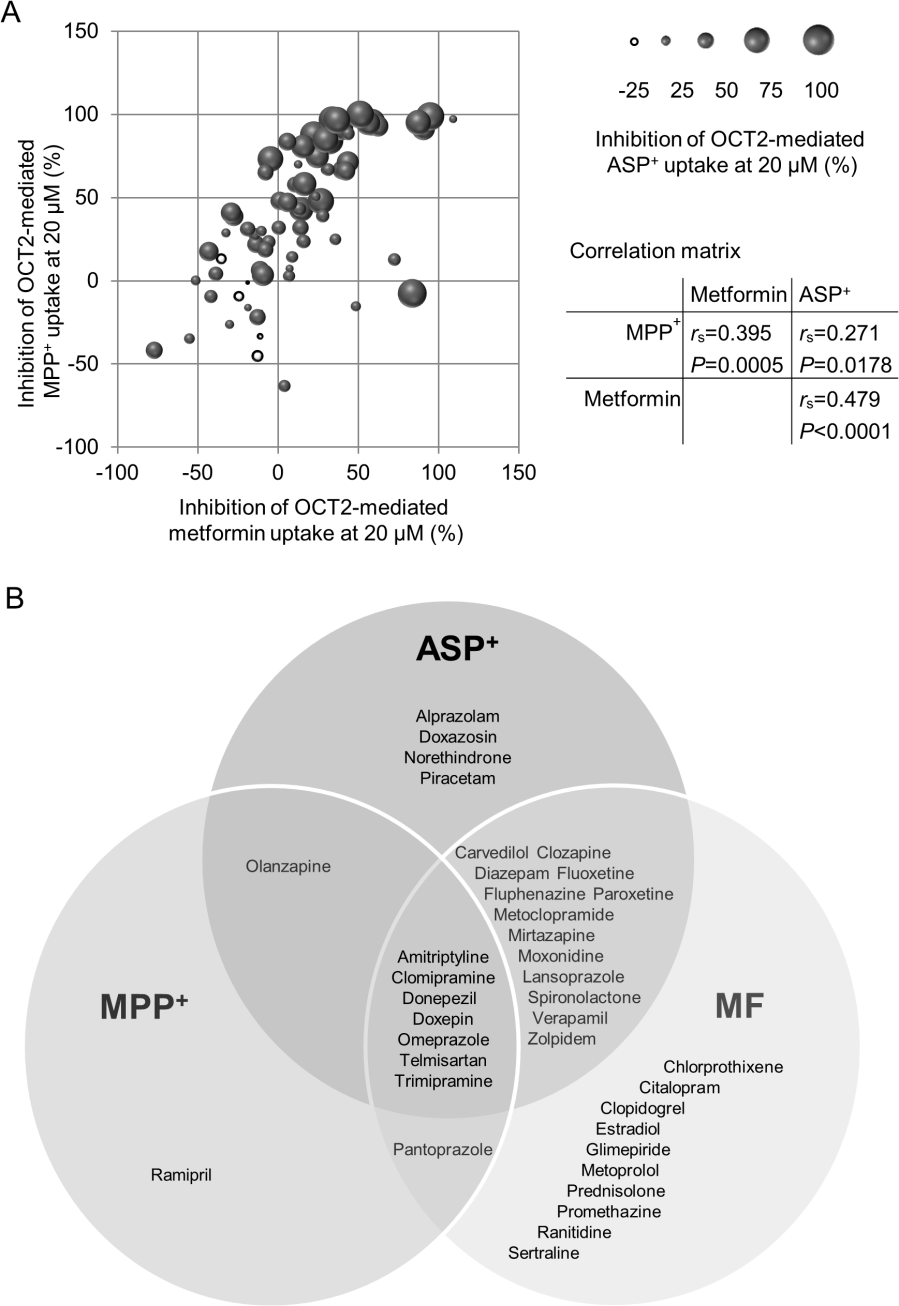
Correlation between inhibition of OCT2-mediated metformin, MPP^+^, and ASP^+^ uptake in HEK-OCT2 cells. (A) Shown are drugs which were tested for each of the three substrates (n = 75). Drugs were tested at 20 μM. The size of the balls represents the inhibition of ASP^+^ uptake. Black balls, positive values; white balls, negative values. ASP^+^ data are from Kido *et al*. (2011). (B) Venn diagram showing unique and common / overlapping inhibitors of metformin, MPP^+^, and ASP^+^ uptake, respectively, by the human OCT2 transporter. Shown are only those drugs which were tested for each of the three substrates (n = 75) and which inhibit uptake of the respective substrate by more than 50%.

We also included additional data with ASP^+^ as the transported substrate, published by Kido *et al*. [7]. ASP^+^ data were available for 75 out of the 125 compounds included in our drug library. Fig 4A shows the correlation between the inhibition of OCT2-mediated metformin, MPP^+^, and ASP^+^ uptake. 37 drugs potently inhibited OCT2-mediated uptake of at least one substrate. Drugs were classified as potent inhibitors if OCT2-mediated uptake of the respective substrate was inhibited by more than 50% at 20 μM. The Venn diagram (Fig 4B) illustrates the substrate-specific inhibition, i.e. it shows potent inhibitors of either one, two or all three substrates. The group of drugs that inhibited the uptake of all three substrates (i.e., metformin, MPP^+^, and ASP^+^), classified as ‘universal’ OCT2 inhibitors, included amitriptyline, clomipramine, donepezil, doxepin, omeprazole, telmisartan, and trimipramine (Fig 4B).

### Predictive Value of Molecular Descriptors

Multiple linear regressions were calculated to predict the degree of OCT2-dependent MPP^+^ or metformin uptake based upon the molecular descriptors TPSA, number of aromatic rings, net charge (at pH 7.4), logD (at pH 7.4), and molecular weight. Additional analyses were performed to ensure there was no collinearity between the explanatory variables. Significant regression equations were found. The models included TPSA, the number of aromatic rings and the net charge as explanatory variables. For inhibition of OCT2-dependent MPP^+^ transport, F (which is the mean square _regression_ divided by the mean square _residual_) was 8.5 (F-statistics *P* = 0.000036) with an R^2^ of 0.17. For inhibition of OCT2-dependent metformin transport, F was 27.7 (*P* < 0.0000001) with an R^2^ of 0.41.

### Clinical Significance of OCT2-Mediated Drug–Drug Interactions

**Fig 5:**
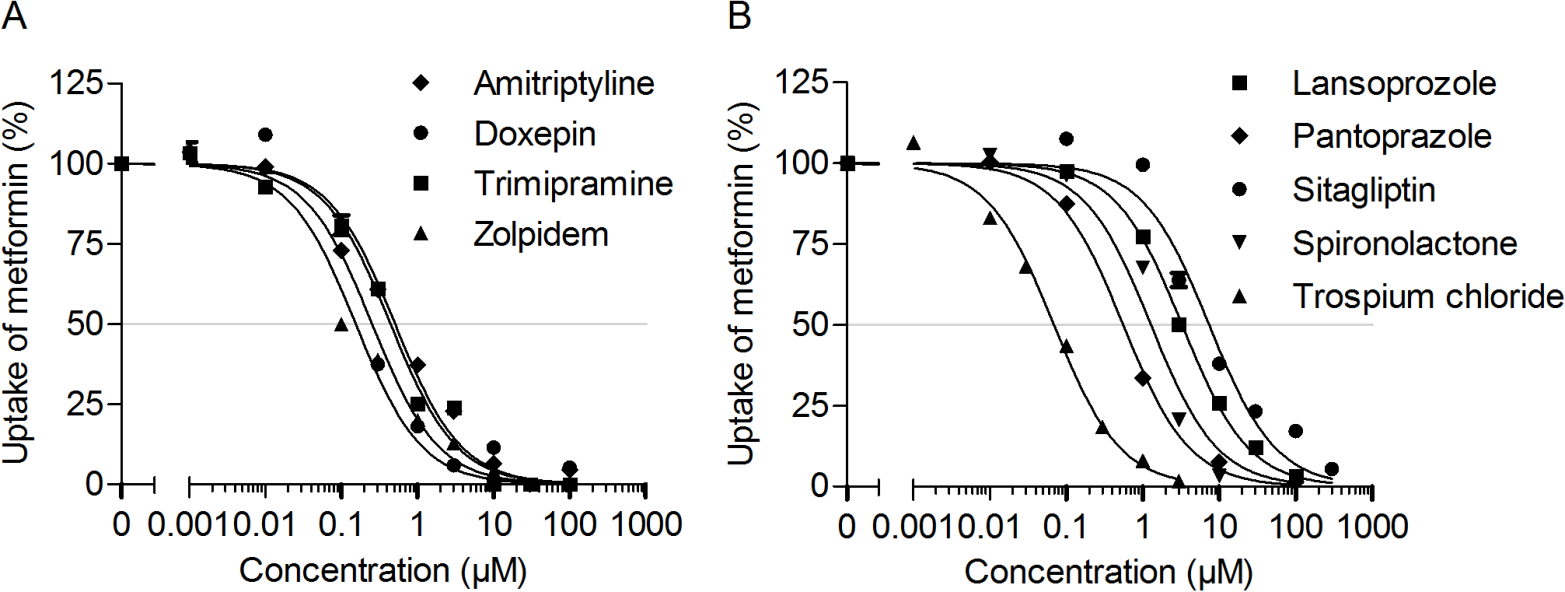
Concentration-dependent inhibition of OCT2-mediated metformin uptake into HEK-OCT2 cells. Shown is the inhibition of metformin uptake by psychoactive (A) and non-psychoactive drugs (B). Data are presented as the mean ± standard error (at least three experiments each on two separate days, i.e., *n* = 6).

Determination of *in vitro* IC_50_ values and comparison with unbound C_max_ values (C_max,u_) achieved in patients are recommended by the International Transporter Consortium and the FDA as a criterion whether a drug might be a clinically relevant OCT2 inhibitor and therefore should be evaluated as an inhibitor in man [14, 15]. **The respective c**_**max**_ **values were obtained from Regenthal *et al*. [16].** For this purpose we determined the *in vitro* IC_50_ values of the 7 most potent inhibitors of OCT2-mediated metformin uptake (Fig 5). We also included lansoprazole and sitagliptin, which belonged to the 20 most potent inhibitors of OCT2-dependent metformin uptake and had relatively high C_max,u_ values [16]. Based on the experimentally determined IC_50_ values the C_max,u_ / IC_50_ ratios were calculated, which were highest for the tricyclic antidepressant doxepin and the proton pump inhibitors lansoprazole and pantoprazole (Table 1).

**Table 1:** Comparison of IC_50_ values for inhibition of OCT2-dependent uptake of metformin and maximum therapeutic plasma concentrations in humans.

OCT2 inhibitor	C_max_ (μM)	C_max,u_ (μM)	IC_50_ (μM)	C_max,u_ / IC_50_
Amitriptyline	1.10	0.04	0.51	0.08
Doxepin	1.30	0.25	0.25	1.00
Lansoprazole	6.00	1.20	3.28	0.37
Pantoprazole	11.4	0.23	0.56	0.41
Sitagliptin	2.30	1.50	7.41	0.20
Spironolactone	1.20	0.02	1.29	0.02
Trimipramine	0.61	0.06	0.44	0.14
Trospium chloride	0.03	0.01	0.07	0.14
Zolpidem	0.65	0.05	0.15	0.33

C_max_, maximum steady-state plasma concentration; C_max,u_, C_max_ of unbound drug; IC_50_, experimentally determined concentration for half maximal inhibition of OCT2-mediated metformin (1000 μM) uptake. C_max_ values were obtained from Regenthal *et al*. [16].

## Discussion

Experimental OCT2 probe substrates, such as MPP^+^ or ASP^+^, have widely been used to identify OCT2 inhibitors [7, 8]. Here we used for the first time not only MPP^+^ but also the clinically used drug metformin as a substrate in an *in vitro* screening approach for the identification of OCT2 inhibitors among a library of commonly prescribed drugs. Metformin was chosen not only because it is a known substrate of OCTs but also because it was recommended by the International Transporter Consortium as a model substrate in clinical drug-drug interaction studies [14].

In this *in vitro* test we identified new potent inhibitors of OCT2-mediated transport of metformin (> 85% inhibition), such as the tricyclic antidepressants clomipramine, doxepin, opipramol and trimipramine, the synthetic opioid methadone, the acetylcholinesterase inhibitor donepezil, the typical antipsychotic fluphenazine, the benzodiazepine oxazepam, and the nonbenzodiazepine hypnotic zolpidem. The steroidal antimineralocorticoid spironolactone, the synthetic hormone drospirenone, the alpha blocker tamsulosin, the angiotensin receptor antagonist telmisartan, and the muscarinic antagonist trospium chloride were also determined as potent inhibitors (> 85% inhibition) of OCT2-mediated metformin uptake. Indeed, doxepin, trimipramine, and trospium chloride were identified as potent OCT2-inhibitors before, however the respective studies did not use metformin but MPP^+^ as the probe substrate [8, 17, 18]. We confirmed previous findings demonstrating that amitriptyline, lansoprazole, omeprazole, pantoprazole, sertraline and sitagliptin were potent inhibitors of OCT2-mediated metformin transport with IC_50_ values in a low micromolar range [19–21].

Using our own and previously published experimental data, we directly compared the inhibition profiles of a set of drugs for the structurally distinct OCT2 substrates metformin, MPP^+^ and ASP^+^. One important finding is that the profiles of OCT2 inhibition showed a moderate but significant correlation between the substrates MPP^+^, ASP^+^, and metformin. Particularly the rather weak correlation between the structurally less diverse substrates MPP^+^ and ASP^+^ (Tanimoto similarity between MPP^+^ and ASP^+^ *T* = 0.28, between metformin and ASP^+^ and metformin and MPP^+^ *T* = 0.01) is surprising. Although inhibition profiles of OCT2-mediated MPP^+^ and ASP^+^ uptake were derived from two independent studies, assay conditions were highly comparable between these studies and thus do not explain the differences in the inhibition profiles. Of note, Thévenod *et al*. recently demonstrated marked differences in the IC_50_ values for example of cimetidine when directly comparing the substrates MPP^+^ and ASP^+^, suggesting a strong substrate-dependence in the inhibitor affinities [22]. The data are consistent with inhibitor-substrate interactions at several structurally distinct sites of the OCT2 protein [23, 24]. Indeed, the physiological role of the renal OCT2 transporter requires that it interacts effectively with a multitude of structurally diverse compounds, a characteristic that is, arguably, inconsistent with the existence of a single site for substrate/inhibitor interactions [23]. Belzer *et al*. also demonstrated the substrate-dependent inhibition of OCT2 and concluded that the development of predictive models of drug-drug interactions with OCT2 must take into account the substrate dependence of ligand interactions with OCT2 [10].

The mode of inhibition has been evaluated in previous studies. Different types of inhibition, e.g., competitive, non-competitive, uncompetitive, and mixed-type inhibition have been identified [9, 25]. Moreover, based on their structural features (pharmacophores), OCT2-inhibitors have been clustered into groups which were associated with specific types of inhibition, such as competitive or non-competitive inhibition [9, 25].

In addition to the pharmacophore-based approach, other studies tried to identify molecular descriptors suitable to discriminate between OCT2 inhibitors and non-inhibitors using the quantitative structure-activity relationship (QSAR) approach [7, 8, 13]. Based on the previous literature we selected a set of five descriptors, which were included as independent variables in multiple linear regression analyses with the inhibition of OCT2-dependent MPP^+^ and metformin uptake as the dependent variables. Irrespective of the substrate, the regression models included TPSA, number of aromatic rings, and net charge as significant predictive variables, confirming their general importance [7, 8, 13]. Although lipophilicity (expressed as logP or logD) has been described before as an important physicochemical feature of OCT2 inhibitor compounds [7, 8, 13], logD was not included in our regression models due to its significant collinearity with TPSA. The predictive value of the molecular descriptors obviously depends on the OCT2 substrate, because the proportion of variance in the dependent variable (i.e., inhibition of OCT2-mediated substrate uptake) which can be explained by the molecular descriptors substantially differed between the substrates metformin (R^2^ = 0.41) and MPP^+^ (R^2^ = 0.17).

Because OCT2-mediated drug-drug interactions are dose dependent, it is imperative to evaluate whether the unbound plasma concentration (i.e., the unbound C_max_ value) of the inhibitor drug reaches a critical level relative to its K_i_ or IC_50_ value for OCT2 inhibition. In drug development, it is recommended to conduct a clinical drug-drug interaction study if the IC_50_ value obtained in cells expressing OCT2 is less than or equal to 10-fold the unbound C_max_ value in humans [14, 15]. Ten-fold, however, was selected to err on the conservative side and does not set a definite threshold for OCT2-dependent inhibitor interactions.

To assess the clinical significance of renal drug-metformin interactions, the inhibitor potency (i.e., IC_50_) was further quantified for nine drugs which were among the most potent inhibitors of OCT2-mediated metformin transport in our screening assay (Table 1). Comparison of the IC_50_ values with the maximum unbound plasma concentrations in humans revealed that the unbound C_max_ / IC_50_ ratio was more than 0.1 for doxepin, lansoprazole, pantoprazole, sitagliptin, trimipramine, trospium chloride, and zolpidem.

Four of these drugs, namely sitagliptin, trospium chloride, lansoprazole, and pantoprazole had been tested for clinically relevant pharmacokinetic interactions with metformin in patients or healthy subjects [26–29]. Co-administration of sitagliptin and metformin did not meaningfully alter the steady-state pharmacokinetics of either agent in patients with type 2 diabetes [26]. Trospium chloride coadministration also did not alter metformin renal clearance and steady-state pharmacokinetics in healthy volunteers [27]. Lansoprazole modestly increased C_max_ and AUC of metformin by 15 and 17%, respectively and decreased its renal clearance by 13% [28]. Similarly, the AUC and C_max_ for metformin was 15% greater following coadministration with pantoprazole [29]. At least in healthy volunteers, coadministration of the proton pump inhibitors had no effect on the maximum glucose level and the area under the serum glucose concentration-time curve, suggesting that the minor changes in pharmacokinetics will not be pharmacodynamically significant. Thus, OCT2-dependent pharmacokinetic interactions with renal clearance of metformin are less likely for drugs with an C_max,u_ / IC_50_ ratio of < 0.2, such as trospium chloride or sitagliptin and start to become obvious with an C_max,u_ / IC_50_ ratio of ∼ 0.4 and greater.

Complete block of tubular metformin transport would decrease renal clearance (CL_R_) of metformin by a maximum of 73%, i.e., the fraction of CL_R_ that is tubular secreted. For comparison, the maximum inhibition of metformin CL_R_ in clinical drug-drug interaction studies was between 37% and 35% for the OCT2-inhibitors pyrimethamine, trimethoprim, and cimetidine [30–33]. For correct interpretation of the *in vivo* findings, it is important to note that OCT2 inhibitors may also be inhibitors of MATEs, which are localized at the apical membrane of proximal tubule cells, and clinical inhibition interactions with metformin may involve MATE1 or MATE2-K in addition to, or even instead of, OCT2 [14]. Cimetidine, for example, a rather weak inhibitor of OCT2 shows higher affinity for MATEs than for OCT2, suggesting that inhibition of the luminal efflux by MATEs, but not basolateral uptake by OCT2, is the likely mechanism underlying the clinically relevant pharmacokinetic drug-drug interactions caused by cimetidine in the kidney [34–36]. The inhibitory interaction of pyrimethamine with renal secretion of metformin has also been attributed primarily to inhibition of MATE1 and MATE2K rather than inhibition of OCT2 [32].

While several drugs inhibited OCT2-mediated substrate uptake or had no effect, we also noted significantly enhanced substrate uptake in the presence of some other drugs. “Negative” inhibition, i.e., transporter stimulation *in vitro* has already been observed by others (Kido *et al*., 2011), although the mechanism is not understood. Even if stimulation would occur *in vivo*, we consider transporter inhibition as considerably more critical since it leads to reduced renal drug secretion and thus potentially drug toxicity.

Our findings confirm the hypothesis that inhibition of OCT2 by clinically used drugs is strongly substrate-dependent. Therefore it is important to conduct *in vitro* inhibition assays with the respective clinically used OCT2—substrates (e.g. metformin) for the prediction of potential drug-drug interactions *in vivo*. The observed substrate dependence of inhibitor affinities also highlights a limitation of previous pharmacophore and structure activity models that were developed to predict *in silico* OCT2-mediated drug-drug interactions of new molecular entities during drug development [7, 23]. These models were based on measurements of the inhibition of transport of experimental substrates by various inhibitors. According to our data it is not very likely that these models are suitable to predict interactions for example with metformin.

## Supporting Information

S1 Fig Inhibition of OCT2-mediated metformin uptake by 125 commonly prescribed drugs at a concentration of 200 μM. Each bar represents one compound tested in HEK-OCT2 cells at a concentration of 200 μM. Data are presented as the mean ± standard error (at least two experiments each on two or more separate days, i.e., n = 4–15); ASS, acetylsalicylic acid.
(TIF)

S2 Fig Inhibition of OCT2-mediated MPP^+^ uptake by 125 commonly prescribed drugs at a concentration of 200 μM. Each bar represents one compound tested in HEK-OCT2 cells at a concentration of 200 μM. Data are presented as the mean ± standard error (at least two experiments each on two or more separate days, i.e., n = 4–15); ASS, acetylsalicylic acid.
(TIF)

S1 Table Experimentally determined inhibition of OCT2 by compounds included in the screening library. List of frequently prescribed drugs which were included in the screening library with their Anatomical Therapeutic Chemical (ATC) codes and Chemical Abstracts Service (CAS) numbers and experimentally determined inhibition of OCT2 by these compounds. MF, metformin; ASS, acetylsalicylic acid.
(PDF)
