# Supplementary figures and images for: Substrate-Dependent Inhibition of the Human Organic Cation Transporter OCT2: A Comparison of Metformin with Experimental Substrates
Source: PLoS One. 2015 Sep 1;10(9):e0136451. doi: 10.1371/journal.pone.0136451 (PMC4556614; doi:10.1371/journal.pone.0136451)

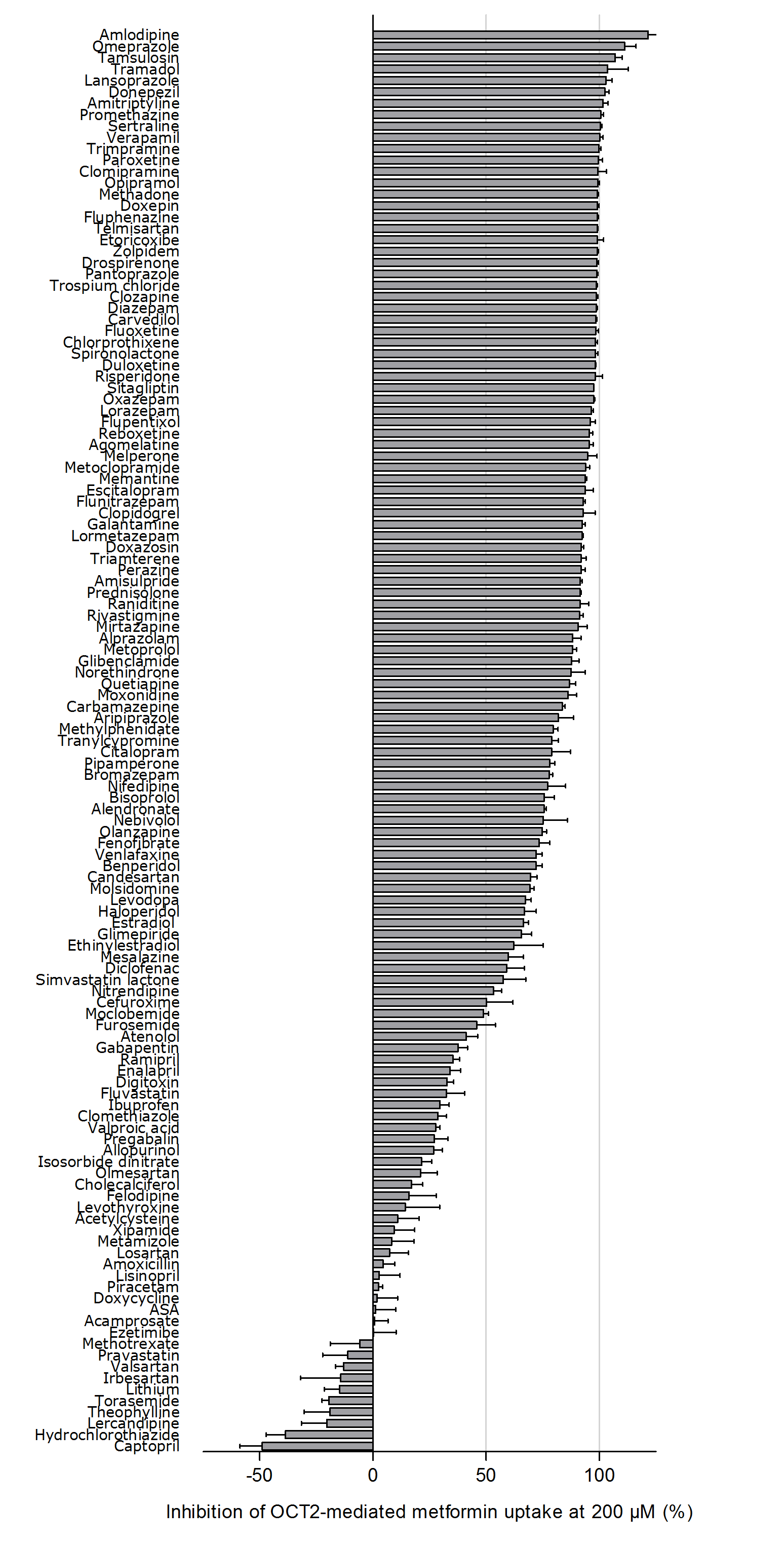

Supplement: S1 Fig — Each bar represents one compound tested in HEK-OCT2 cells at a concentration of 200 μM. Data are presented as the mean ± standard error (at least two experiments each on two or more separate days, i.e., n = 4–15); ASS, acetylsalicylic acid. (TIF) [file pone.0136451.s001.tif]

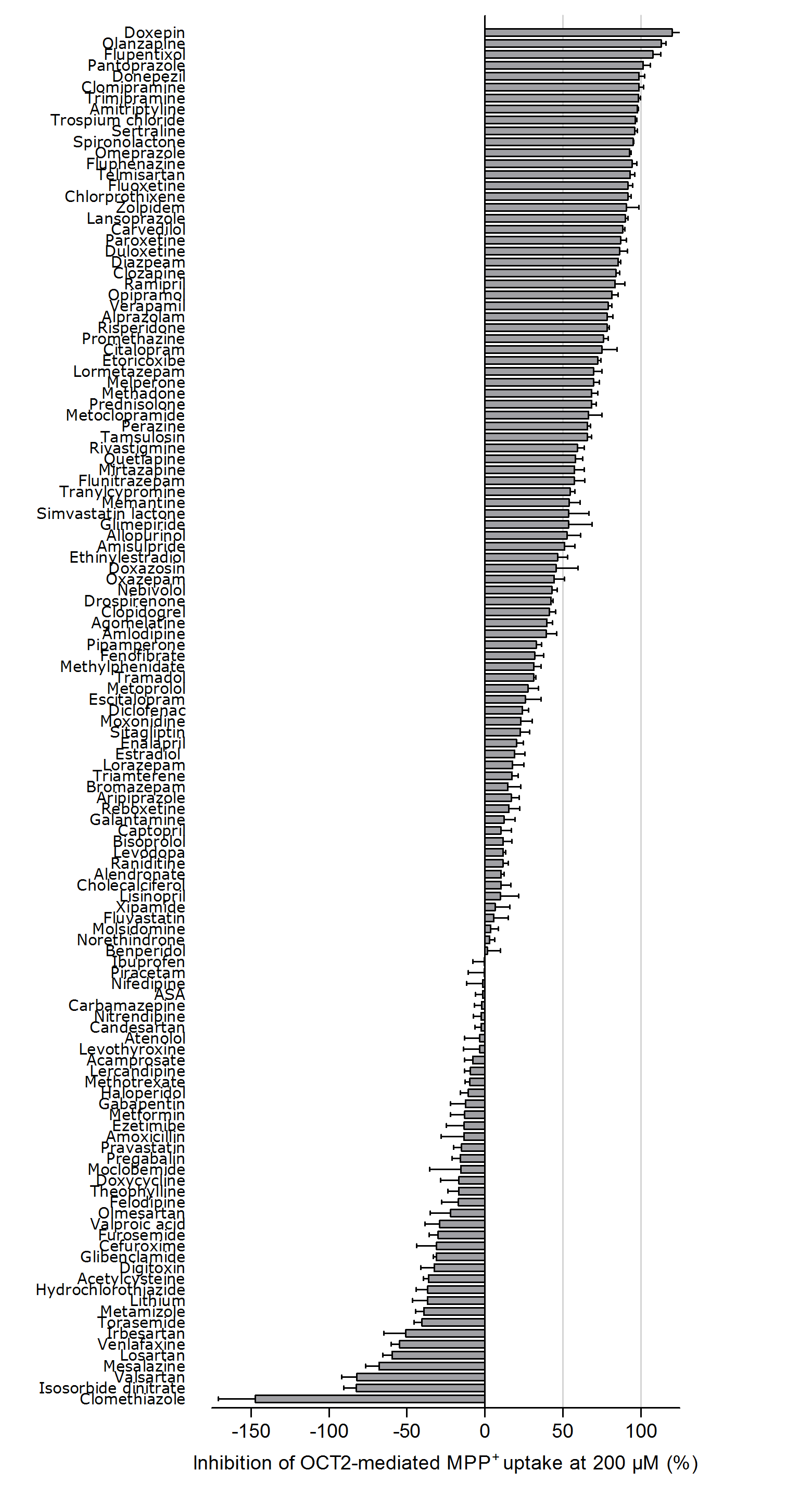

Supplement: S2 Fig — Each bar represents one compound tested in HEK-OCT2 cells at a concentration of 200 μM. Data are presented as the mean ± standard error (at least two experiments each on two or more separate days, i.e., n = 4–15); ASS, acetylsalicylic acid. (TIF) [file pone.0136451.s002.tif]
