# Supplementary material for: Substrate-Dependent Inhibition of the Human Organic Cation Transporter OCT2: A Comparison of Metformin with Experimental Substrates
Source: PLoS One. 2015 Sep 1;10(9):e0136451. doi: 10.1371/journal.pone.0136451 (PMC4556614; doi:10.1371/journal.pone.0136451)
Supplement: S1 Table — List of frequently prescribed drugs which were included in the screening library with their Anatomical Therapeutic Chemical (ATC) codes and Chemical Abstracts Service (CAS) numbers and experimentally determined inhibition of OCT2 by these compounds. MF, metformin; ASS, acetylsalicylic acid. (PDF) [file pone.0136451.s003.pdf]

Table S1

Drug	ATC code	CAS number	% Inhibition of OCT2-dependent MF uptake		% Inhibition of OCT2-dependent MPP^+ uptake	
			at 20 µM (mean ± SEM)	at 200 µM (mean ± SEM)	at 20 µM (mean ± SEM)	At 200 µM (mean ± SEM)
Acamprosate	N07BB03	77337-73-6	2.7 ± 4.8	0.9 ± 5.8	-8.1 ± 4.4	-7.7 ± 5.2
Acetylcysteine	R05CB01, V03AB23, S01XA08	616-91-1	13.2 ± 6.3	11.0 ± 9.4	-35.6 ± 7.7	-36.0 ± 3.2
Agomelatine	N06AX22	138112-76-2	71.3 ± 2.6	95.5 ± 1.8	-10.3 ± 2.7	39.9 ± 3.5
Alendronate	M05BA04	66376-36-1	30.7 ± 9.0	75.6 ± 0.5	5.4 ± 3.9	10.5 ± 2.0
Allopurinol	M04AA01	315-30-0	24.8 ± 6.9	26.8 ± 3.9	35.6 ± 7.5	52.7 ± 8.8
Alprazolam	N05BA12	28981-97-7	43.0 ± 5.0	88.3 ± 3.6	14.3 ± 7.4	78.5 ± 3.6
Amisulpride	N05AL05	71675-85-9	52.0 ± 2.1	91.7 ± 0.7	25.5 ± 7.0	51.3 ± 6.7
Amitriptyline	N06AA09	549-18-8	95.4 ± 4.8	101.7 ± 2.2	58.4 ± 2.8	97.8 ± 0.6
Amlodipine	C08CA01	88150-42-9	21.9 ± 9.0	110.0 ± 17.3	-13.8 ± 7.4	39.3 ± 6.8
Amoxicillin	J01CA04	26787-78-0	3.2 ± 6.6	4.5 ± 5.1	-21.0 ± 11.1	-13.5 ± 14.5
Aripiprazole	N05AX12	129722-12-9	53.4 ± 9.1	82.0 ± 6.7	6.7 ± 3.6	16.9 ± 5.2
ASA	N02BA01, B01AC06, A01AD05	50-78-2	-18.7 ± 11.0	1.3 ± 8.9	-10.6 ± 5.7	-1.5 ± 4.5
Atenolol	C07AB03	29122-68-7	28.0 ± 2.4	41.1 ± 5.1	-14.9 ± 3.5	-3.2 ± 9.7
Benperidol	N05AD07	2062-84-2	58.7 ± 3.3	72.2 ± 2.7	-21.5 ± 2.8	1.6 ± 8.3
Bisoprolol	C07AB07	66722-44-9	57.2 ± 4.0	75.8 ± 4.5	-16.1 ± 2.2	11.8 ± 5.5
Bromazepam	N05BA08	1812-30-2	27.6 ± 2.6	77.9 ± 1.4	-16.3 ± 9.0	14.7 ± 8.3
Candesartan	C09CA06	139481-59-7	48.0 ± 3.7	69.7 ± 2.7	0.9 ± 0.1	-2.4 ± 4.0
Captopril	C09AA01	62571-86-2	-21.6 ± 19.5	-59.0 ± 0.9	3.8 ± 5.7	10.2 ± 6.8
Carbamazepine	N03AF01	298-46-4	0.2 ± 3.8	83.8 ± 1.2	-51.4 ± 9.2	-2.1 ± 4.4
Carvedilol	C07AG02	72956-09-3	84.3 ± 5.2	98.6 ± 0.5	32.2 ± 3.7	88.5 ± 1.5
Cefuroxime	J01DC02	55268-75-2	29.9 ± 9.7	50.3 ± 11.6	-10.5 ± 7.0	-30.8 ± 12.9
Chlorprothixene	N05AF03	6469-93-8	66.8 ± 7.9	98.4 ± 0.9	29.9 ± 7.5	91.7 ± 2.2

Cholecalciferol	A11CC05	67-97-0	2.8 ± 15.7	17.1 ± 5.0	6.8 ± 5.1	10.4 ± 6.1
Citalopram	N06AB04	59729-32-7	66.6 ± 7.9	78.9 ± 8.4	40.0 ± 9.9	75.2 ± 9.7
Clomethiazole	N05CM02	6001-74-7	-9.2 ± 6.8	28.7 ± 3.9	-6.7 ± 9.2	-147.3 ± 23.7
Clomipramine	N06AA04	17321-77-6	91.5 ± 4.9	99.5 ± 3.7	90.3 ± 1.5	98.8 ± 3.0
Clopidogrel	B01AC04	120202-66-6	70.0 ± 2.0	92.8 ± 5.4	12.5 ± 0.8	41.5 ± 4.0
Clozapine	N05AH02	5786-21-0	71.4 ± 1.6	98.7 ± 0.7	43.4 ± 4.7	84.1 ± 2.5
Diazepam	N05BA01	439-14-5	83.3 ± 2.0	98.7 ± 0.5	32.2 ± 9.2	85.6 ± 1.4
Diclofenac	M01AB05, M02AA15, S01BC03, D11AX18	15307-86-5	14.3 ± 8.8	59.1 ± 6.5	8.8 ± 8.9	24.0 ± 4.0
Digitoxin	C01AA04., C01AA54, C05BZ05	71-63-6	28.7 ± 7.9	32.8 ± 2.9	-32.6 ± 3.8	-32.4 ± 8.5
Donepezil	N06DA02	884740-09-4	92.9 ± 1.2	102.5 ± 1.8	62.2 ± 8.1	99.0 ± 3.4
Doxazosin	C02CA04	74191-85-8	48.0 ± 5.5	92.1 ± 1.0	26.7 ± 10.3	45.8 ± 14.0
Doxepin	N06AA12	1229-29-4	98.8 ± 2.1	99.3 ± 0.7	94.7 ± 3.9	107.6 ± 5.1
Doxycycline	J01AA02, A01AB22	564-25-0	-21.8 ± 1.9	1.9 ± 9.1	-12.9 ± 13.1	-16.6 ± 11.7
Drospirenone	G03AA12, G03FA17	67392-87-4	88.0 ± 7.3	99.1 ± 0.7	0.0 ± 3.6	42.6 ± 1.4
Duloxetine	N06AX21	136434-34-9	76.5 ± 0.9	98.3 ± 0.2	23.3 ± 6.0	86.4 ± 5.1
Enalapril	C09AA02	75847-73-3	7.5 ± 2.3	34.2 ± 4.5	7.1 ± 7.4	20.2 ± 5.6
Escitalopram	N06AB10	128196-01-0	62.6 ± 9.2	93.8 ± 3.6	-26.3 ± 7.9	26.0 ± 10.1
Estradiol	G03CA03	50-28-2	57.9 ± 5.4	66.6 ± 2.1	10.6 ± 4.2	19.0 ± 6.5
Ethinylestradiol	G03CA01, L02AA03	57-63-6	31.8 ± 8.1	62.2 ± 13.1	14.0 ± 8.8	46.8 ± 6.3
Etoricoxib	M01AH05	202409-33-4	64.1 ± 6.8	99.2 ± 2.7	2.3 ± 4.0	72.4 ± 2.2
Ezetimibe	C10AX09	163222-33-1	-6.8 ± 9.7	0.2 ± 10.0	-12.6 ± 12.4	-13.2 ± 11.5
Felodipine	C08CA02	72509-76-3	4.9 ± 6.1	15.9 ± 12.1	-8.8 ± 12.0	-16.8 ± 10.8
Fenofibrate	C10AB05	49562-28-9	43.0 ± 9.9	73.4 ± 4.7	12.8 ± 5.0	32.0 ± 5.7
Flunitrazepam	N05CD03	1622-62-4	74.1 ± 3.1	92.9 ± 0.9	5.1 ± 8.4	57.5 ± 6.6
Fluoxetine	N06AB03	56296-78-7	79.0 ± 0.9	98.5 ± 1.2	20.2 ± 3.5	91.8 ± 2.9
Flupentixol	N05AF01	51529-01-2	75.0 ± 7.9	96.1 ± 2.1	22.9 ± 7.5	107.8 ± 5.0
Fluphenazine	N05AB02	146-56-5	91.4 ± 0.9	99.3 ± 0.4	39.3 ± 7.0	94.3 ± 3.3

Fluvastatin	C10AA04	93957-54-1	-21.1 ± 10.3	32.5 ± 8.1	-10.1 ± 6.1	5.8 ± 9.1
Furosemide	C03CA01	54-31-9	31.1 ± 7.1	45.8 ± 8.3	-19.1 ± 7.8	-30.0 ± 5.8
Gabapentin	N03AX12	60142-96-3	9.1 ± 8.2	37.6 ± 4.2	-8.4 ± 9.6	-12.5 ± 9.4
Galantamine	N06DA04	1953-04-4	51.5 ± 2.8	92.5 ± 1.2	-11.7 ± 9.4	12.3 ± 7.0
Glibenclamide	A10BB01	10238-21-8	38.7 ± 11.9	87.9 ± 3.2	-27.7 ± 5.0	-31.1 ± 1.7
Glimepiride	A10BB12	93479-97-1	66.8 ± 2.9	65.6 ± 4.6	31.1 ± 7.6	53.8 ± 15.2
Haloperidol	N05AD01	52-86-8	6.4 ± 8.9	66.9 ± 5.3	-11.2 ± 5.0	-10.6 ± 5.1
Hydrochlorothiazide	C03AA03	58-93-5	-34.7 ± 16.9	-38.6 ± 8.5	-55.4 ± 10.2	-36.6 ± 7.4
Ibuprofen	M02AA13, M01AE01, C01EB16, G02CC01	15687-27-1	23.4 ± 6.7	29.6 ± 4.1	-5.8 ± 8.0	-0.3 ± 7.5
Irbesartan	C09CA04	138402-11-6	-16.2 ± 14.4	-14.2 ± 17.8	-18.9 ± 12.4	-50.7 ± 14.1
Isosorbide dinitrate	C01DA08, C01DA58	87-33-2	-19.5 ± 6.9	21.7 ± 4.4	-20.4 ± 5.0	-82.4 ± 8.0
Lansoprazole	A02BC03	103577-45-3	87.7 ± 1.6	103.0 ± 2.7	22.1 ± 5.3	90.1 ± 1.8
Lercanidipine	C08CA13	100427-26-7	7.8 ± 3.8	-20.4 ± 11.1	-13.3 ± 8.6	-9.3 ± 3.6
Levodopa	N04BA01	59-92-7	-9.3 ± 6.8	67.3 ± 2.6	-24.6 ± 0.5	11.8 ± 1.4
Levothyroxine	H03AA01	51-48-9	0.8 ± 9.6	14.3 ± 15.2	3.6 ± 4.1	-3.3 ± 10.3
Lisinopril	C09AA03	83915-83-7	-10.3 ± 12.9	2.9 ± 9.0	16.0 ± 9.0	9.8 ± 11.8
Lithium	N05AN01	7447-41-8	-15.2 ± 7.9	-14.7 ± 6.8	-28.4 ± 7.5	-36.7 ± 9.7
Lorazepam	N05BA06	846-49-1	32.0 ± 2.2	96.5 ± 1.0	0.3 ± 5.1	17.7 ± 7.3
Lormetazepam	N05CD06	848-75-9	77.0 ± 0.6	92.4 ± 0.5	47.8 ± 8.4	69.8 ± 5.3
Losartan	C09CA01	114798-26-4	-9.3 ± 10.4	7.4 ± 8.2	-41.8 ± 8.1	-59.5 ± 6.1
Melperone	N05AD03	1622-79-3	75.0 ± 3.4	94.9 ± 4.2	8.6 ± 10.1	69.7 ± 3.8
Memantine	N06DX01	41100-52-1	54.0 ± 2.1	93.9 ± 0.6	20.6 ± 8.6	54.0 ± 7.0
Mesalazine	A07EC02	89-57-6	21.6 ± 11.1	59.8 ± 6.7	-51.3 ± 6.1	-68.0 ± 8.5
Metamizole	N02BB02	68-89-3	-4.8 ± 6.4	8.4 ± 9.8	-31.6 ± 6.0	-39.1 ± 5.1
Metformin	A10BA02	657-24-9			-17.2 ± 10.1	-12.9 ± 8.4
Methadone	N07BC02	1095-90-5	94.6 ± 0.7	99.3 ± 0.3	8.9 ± 4.4	68.7 ± 3.8
Methotrexate	L01BA01, L04AX03	59-05-2	-1.1 ± 11.4	-5.8 ± 13.0	-19.0 ± 7.2	-9.5 ± 3.0
Methylphenidate	N06BA04	298-59-9	17.9 ± 4.6	79.6 ± 2.3	11.5 ± 8.9	31.6 ± 4.7

Metoclopramide	A03FA01	364-62-5	74.7 ± 8.5	93.9 ± 0.6	24.5 ± 2.9	66.6 ± 8.4
Metoprolol	C07AB02	37350-58-6	50.5 ± 5.9	88.2 ± 1.7	23.2 ± 6.5	27.5 ± 6.9
Mirtazapine	N06AX11	85650-52-8	73.3 ± 3.0	90.7 ± 3.9	-4.8 ± 4.0	57.6 ± 6.1
Moclobemide	N06AG02	71320-77-9	4.3 ± 2.8	48.9 ± 2.1	-38.7 ± 8.5	-16.1 ± 17.7
Molsidomine	C01DX12	25717-80-0	41.4 ± 3.6	69.5 ± 1.7	-1.7 ± 8.3	3.8 ± 4.9
Moxonidine	C02AC05	75438-57-2	58.1 ± 14.5	86.1 ± 2.9	16.3 ± 6.5	23.1 ± 7.1
Nebivolol	C07AB12	118457-14-0	61.4 ± 10.4	75.2 ± 10.8	-13.7 ± 13.3	43.2 ± 3.3
Nifedipine	C08CA05	21829-25-4	18.8 ± 4.4	77.1 ± 8.0	-8.1 ± 6.1	-1.4 ± 10.2
Nitrendipine	C08CA08	39562-70-4	0.4 ± 9.8	53.2 ± 3.7	4.0 ± 6.3	-2.4 ± 5.0
Norethindrone	G03AC01, G03DC02	68-22-4	41.0 ± 11.0	87.7 ± 6.1	-29.4 ± 6.4	3.0 ± 3.3
Olanzapine	N05AH03	132539-06-1	-7.5 ± 5.7	74.9 ± 1.8	83.5 ± 9.4	109.7 ± 2.8
Olmesartan	C09CA08, C09DA08	144689-24-7	-0.1 ± 10.9	21.1 ± 7.3	-23.8 ± 7.1	-21.9 ± 13.3
Omeprazole	A02BC01	73590-58-6	94.9 ± 2.4	107.4 ± 4.3	56.0 ± 4.0	93.0 ± 0.9
Opipramol	N06AA05	909-39-7	94.7 ± 1.5	99.5 ± 0.7	14.0 ± 7.6	81.6 ± 3.9
Oxazepam	N05BA04	604-75-1	88.1 ± 1.2	97.5 ± 0.5	37.6 ± 4.7	44.6 ± 6.5
Pantoprazole	A02BC02	102625-70-7	97.1 ± 1.4	98.9 ± 0.6	108.9 ± 4.0	101.7 ± 4.4
Paroxetine	N06AB05	110429-35-1	80.6 ± 3.1	99.6 ± 1.9	15.5 ± 3.8	87.2 ± 3.6
Perazine	N05AB10	84-97-9	53.6 ± 3.6	92.0 ± 1.8	58.5 ± 2.8	65.9 ± 2.0
Pipamperone	N05AD05	2448-68-2	33.9 ± 6.0	78.1 ± 2.2	12.0 ± 4.5	33.1 ± 3.4
Piracetam	N06BX03	7491-74-9	3.4 ± 3.4	2.5 ± 1.7	-9.5 ± 12.1	-0.5 ± 10.0
Pravastatin	C10AA03	81093-37-0	-26.3 ± 11.1	-11.1 ± 11.1	-30.2 ± 5.5	-15.0 ± 5.2
Prednisolone	A07EA01, C05AA04, D07AA03, D07XA02, H02AB06, R01AD02, S01BA04, S01CB02, S02BA03, S03BA02	50-24-8	66.0 ± 2.5	91.5 ± 0.5	42.1 ± 4.4	68.6 ± 2.8
Pregabalin	N03AX16	148553-50-8	-1.3 ± 9.0	27.2 ± 5.9	-29.6 ± 7.5	-15.8 ± 5.2
Promethazine	D04AA10, R06AD02	58-33-3	83.7 ± 3.3	100.7 ± 1.3	5.7 ± 3.8	76.0 ± 3.0

Quetiapine	N05AH04	111974-72-2	23.7 ± 5.9	86.8 ± 2.8	15.8 ± 9.1	58.3 ± 4.5
Ramipril	C09AA05	87333-19-5	12.9 ± 4.1	35.4 ± 2.9	72.5 ± 7.4	83.6 ± 6.2
Ranitidine	A02BA02	66357-35-5	65.2 ± 7.7	91.5 ± 3.8	-7.9 ± 12.9	11.6 ± 3.4
Reboxetine	N06AX18	98769-84-7	64.8 ± 4.0	95.6 ± 1.4	-21.6 ± 8.3	15.4 ± 8.0
Risperidone	N05AX08	106266-06-2	81.0 ± 4.4	98.3 ± 3.1	57.8 ± 5.2	78.4 ± 1.5
Rivastigmine	N06DA03	123441-03-2	51.8 ± 4.0	91.4 ± 1.5	25.1 ± 9.1	59.4 ± 4.6
Sertraline	N06AB06	79559-97-0	88.4 ± 2.1	100.5 ± 0.7	43.6 ± 2.9	96.1 ± 1.7
Simvastatin lactone	C10AA01, C10BA02	79902–63–9	-15.4 ± 5.5	57.6 ± 10.0	48.5 ± 9.0	53.8 ± 13.1
Sitagliptin	A10BH01	486460-32-6	85.9 ± 1.1	97.5 ± 0.1	-8.2 ± 7.8	22.7 ± 6.0
Spironolactone	C03DA01	52-01-7	96.8 ± 0.5	98.4 ± 1.0	33.5 ± 4.7	95.0 ± 0.5
Tamsulosin	G04CA02	106133-20-4	87.9 ± 0.9	107.0 ± 3.1	14.9 ± 4.8	65.7 ± 2.9
Telmisartan	C09CA07	144701-48-4	95.1 ± 0.7	99.2 ± 0.2	87.1 ± 2.1	93.3 ± 2.8
Theophylline	R03DA04, R03DA54, R03DB04, R03DA74	58-55-9	-21.5 ± 13.0	-36.6 ± 10.9	-12.9 ± 5.7	-16.7 ± 6.8
Torasemide	C03CA04	56211-40-6	-33.2 ± 5.0	-19.5 ± 0.8	-11.4 ± 2.3	-40.3 ± 5.0
Tramadol	N02AX02	36282-47-0	47.1 ± 9.5	103.7 ± 9.2	5.9 ± 7.9	31.4 ± 1.4
Tranylcypromine	N06AF04	13492-01-8	39.0 ± 3.3	79.1 ± 2.9	27.8 ± 5.6	54.7 ± 3.1
Triamterene	C03DB02	396-01-0	48.0 ± 5.1	92.0 ± 2.1	6.6 ± 11.2	17.5 ± 3.8
Trimipramine	N06AA06	521-78-8	99.9 ± 0.5	100.0 ± 0.8	51.0 ± 10.2	98.6 ± 1.2
Trospium chloride	G04BD09	10405-02-4	99.3 ± 0.1	98.8 ± 0.4	83.2 ± 2.2	96.7 ± 0.7
Valproic acid	N03AG01	1069-66-5	18.5 ± 7.6	27.7 ± 1.9	-17.3 ± 9.5	-29.0 ± 9.2
Valsartan	C09CA03, C09DB01, C09DA03	137862-53-4	-41.7 ± 1.8	-12.9 ± 3.6	-77.2 ± 6.0	-82.1 ± 9.9
Venlafaxine	N06AX16	99300-78-4	17.6 ± 3.2	72.2 ± 2.5	-43.3 ± 13.0	-54.3 ± 13.1
Verapamil	C08DA01	52-53-9	84.8 ± 2.8	100.2 ± 1.5	29.4 ± 7.0	79.1 ± 2.3
Xipamide	C03BA10	14293-44-8	-22.3 ± 9.7	9.4 ± 9.2	10.5 ± 3.7	6.6 ± 9.3
Zolpidem	N05CF02	82626-48-0	96.7 ± 0.3	99.1 ± 0.5	36.5 ± 4.3	90.7 ± 8.2
